# Draft Genome Sequence of Bacillus velezensis Strain Marseille-Q1230, Isolated from a Stool Sample from a Severely Malnourished Child

**DOI:** 10.1128/MRA.00514-21

**Published:** 2021-08-19

**Authors:** Salimata Konaté, Rita Zgheib, Aminata Camara, Ogobara Doumbo, Abdoulaye Djimdé, Abdoulaye Kassoum Koné, Mahamadou Ali Théra, Pierre-Edouard Fournier, Maryam Tidjani Alou, Didier Raoult, Matthieu Million

**Affiliations:** a Aix-Marseille Université, IRD, AP-HM, MEPHI, Marseille, France; b Institut Hospitalo-Universitaire Méditerranée Infection, Marseille, France; c Aix-Marseille Université, IRD, AP-HM, SSA, VITROME, Marseille, France; d Malaria Research and Training Center, Faculty of Medicine, Pharmacy, and Odontostomatology, University of Bamako, Bamako, Mali; University of Rochester School of Medicine and Dentistry

## Abstract

Bacillus velezensis, a species first described in 2005, has been mostly associated with plants and the environment. To date, there is no genome available for this species from human samples. In this announcement, we present the genome of Bacillus velezensis strain Marseille-Q1230, which was isolated from a stool sample from a child suffering from severe acute malnutrition. The genome assembled into 15 contigs and had a size of 3,861,152 bp, with a GC content of 46.6%. A total of 3,716 protein-coding genes, including 3 antibiotic resistance genes and 92 RNAs, were predicted.

## ANNOUNCEMENT

The genus *Bacillus*, a member of the phylum *Firmicutes*, includes more than 200 validly published species (https://lpsn.dsmz.de/genus/bacillus) (1). Bacillus velezensis was first isolated by Ruiz-García et al. from the Vélez River in Malaga, Spain (2), and possesses bactericidal (3, 4) and fungicidal (5) abilities, thus promoting plant growth and controlling wheat diseases (6). No previous studies have reported its presence in the human microbiome, as this species has been isolated only from plants or environmental samples (7, 8). Here, we present the genome sequence of a B. velezensis strain that was isolated from a human sample collected as part of a childhood malnutrition study in Mali; the study was approved by the Malian ethical committee (approval number 2014/46/CE/FMPOS). More specifically, B. velezensis strain Marseille-Q1230 was isolated from the stool of a severely malnourished Malian child <5 years of age using the culturomics approach, which is a high-throughput approach consisting of diversification and multiplication of culture conditions followed by rapid identification by matrix-assisted laser desorption ionization–time of flight mass spectrometry (MALDI-TOF MS) (9, 10).

Genomic DNA of strain Marseille-Q1230 was extracted using the EZ1 biorobot (Qiagen) with the EZ1 DNA tissue kit before sequencing using the MiSeq platform (Illumina Inc., San Diego, CA, USA) with the paired-end strategy. The library was prepared following the workflow of the Nextera XT DNA library preparation kit (Illumina) (11). Automated cluster generation and paired-end sequencing with dual index reads were performed in a single 39-hour run (2 × 250 bp); 5.00 Gb of data was obtained from a 521-K/mm^2^ cluster density, with 94.11% of clusters passing the quality control filters. Within this run, the index representation for strain Marseille-Q1230 was assessed as 5.09%. Finally, 10,125,033 paired-end reads were filtered according to read quality using FastQC v0.11.8 (https://www.bioinformatics.babraham.ac.uk/projects/fastqc).

**FIG 1:**
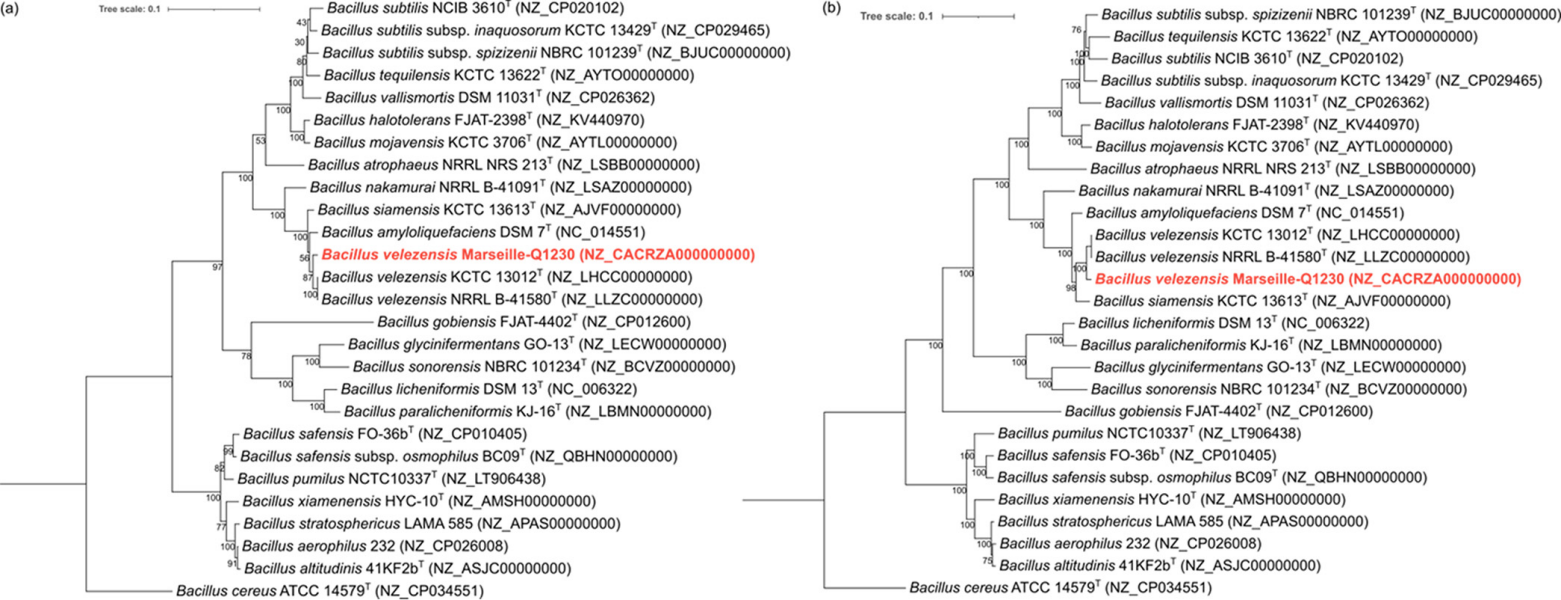
(a) Phylogenetic tree inferred from a comparison of the *rpoB* gene sequences using the maximum likelihood method with 1,000 bootstrap replicates and the Kimura-2 parameter with MEGA X software (19). (b) Core genome-based phylogenetic tree generated from the alignment of 208 core genes for all of the compared strains, including strain Marseille-Q1230 (red). The core gene alignment was generated using Roary v3.13.0 with 80% identity (20). The tree was inferred with FastTree v2.1.10 (21). Genome accession numbers are indicated in parentheses. Type strains are indicated with a superscript T. Numbers at the nodes are bootstrap values. The scale bars indicate 10% sequence divergence.

The resulting forward (113,107,188 bases) and reverse (113,148,534 bases) reads were assembled using SPAdes v3.14.0 (12). FastQC and SPAdes were used with default parameters. Strain Marseille-Q1230 was identified after the 16S rRNA gene sequence was extracted from the genome and matched against the GenBank database using BLASTn (accessed 6 August 2020) (13). Strain Marseille-Q1230 exhibited 16S rRNA gene sequence similarity of >99% to multiple species from the Bacillus subtilis species complex. Because the 16S rRNA gene sequence failed to discriminate within the aforementioned species complex, we also used OrthoANI v0.93.1 software (14), core genome-based and *rpoB*-based phylogeny (15), and *recQ* sequence identities (determined by local BLASTn v2.9.0) (16). Both *rpoB* and *recQ* genes were extracted from the annotated genomes. All of these methods revealed a close relationship between strain Marseille-Q1230 and B. velezensis, as both *recQ* sequence identities and OrthoANI values were higher than the species-delineating thresholds (96.6% for *recQ* and 95% to 96% for OrthoANI) (Table 1 and Fig. 1) (14, 16). Therefore, phylogenetic and genomic analyses identified strain Marseille-Q1230 as B. velezensis.

**TABLE 1:** Pairwise OrthoANI values and *recQ* gene identities between strain Marseille-Q1230 and other type strains from the Bacillus subtilis species complex

Strain^*a*^	OrthoANI value (%) or *recQ* gene identity (%)^*b*^									
	Strain 1	Strain 2	Strain 3	Strain 4	Strain 5	Strain 6	Strain 7	Strain 8	Strain 9	Strain 10
1	100.00	77.33	70.24	97.82	94.47	77.04	86.42	94.07	99.95	77.26
2	75.64	100.00	71.06	77.09	77.41	87.31	77.83	77.51	77.39	80.57
3	66.29	67.18	100.00	70.43	70.37	70.96	70.54	70.48	70.29	70.75
4	98.54	75.36	66.29	100.00	94.35	77.00	86.47	94.05	97.84	77.08
5	95.28	74.56	66.05	95.17	100.00	77.00	86.11	93.98	94.44	77.45
6	75.99	84.84	67.92	75.75	74.70	100.00	77.46	77.07	77.06	79.56
7	85.69	75.42	67.10	85.35	85.13	76.91	100.00	86.82	86.59	78.39
8	94.94	75.87	65.77	94.77	94.44	75.54	85.69	100.00	94.10	77.32
9	100.00	75.64	66.29	98.54	95.28	75.99	85.69	94.94	100.00	77.37
10	77.02	79.77	67.39	76.80	76.37	77.82	78.37	77.19	77.02	100.00

a Strain 1, Bacillus velezensis KCTC 13012^T^; strain 2, Bacillus halotolerans FJAT-2398^T^; strain 3, Bacillus xiamenensis HYC-10^T^; strain 4, Bacillus velezensis Marseille-Q1230; strain 5, Bacillus siamensis KCTC 13613^T^; strain 6, Bacillus subtilis NCIB 3610^T^; strain 7, Bacillus nakamurai NRRL B-41091^T^; strain 8, Bacillus amyloliquefaciens DSM 7^T^; strain 9, Bacillus velezensis NRRL B-41580^T^; strain 10, Bacillus atrophaeus NRRL NRS 213^T^.

b Pairwise OrthoANI values are shown in the upper half of the table and *recQ* gene identity values in the lower half.

Strain Marseille-Q1230 had a genome size of 3,861,152 bp (*N*_50_ value of 583,987 bp) assembled into 15 contigs, with a GC content of 46.6% and 29.3× coverage. Annotation with Prokka v1.13 software (17) predicted 3,716 protein-coding genes and 92 RNA genes (10 rRNA genes, 81 tRNA genes, and 1 transfer-messenger RNA gene). Compared to NCBI Prokaryotic Genome Annotation Pipeline (PGAP) annotation, a few differences were obtained using the Prokka software, probably due to different parameters and databases used by the two tools. Using ABRicate with the NCBI resistance database and an identity cutoff value of 80%, three resistance genes, namely, *rphC* (rifamycin-inactivating phosphotransferase), *tet*(L) (tetracycline efflux major facilitator superfamily [MFS] transporter), and *satA* (streptothricin *N*-acetyltransferase), were detected (18).

### Data availability.

The raw reads and genome sequence of Bacillus velezensis strain Marseille-Q1230 have been deposited in GenBank under accession numbers SRR14270290 and CACRZA000000000, respectively.
